# Acromioclavicular Septic Arthritis Caused by *Veillonella parvula*

**DOI:** 10.1155/2019/7106252

**Published:** 2019-11-27

**Authors:** Marc Prod'homme, Gilles Pfander, Patricia Pavese, Alexandre Cech, Islam Abouelnaga, Lionel Helfer

**Affiliations:** ^1^Orthopaedic Surgery Department, La Tronche, France; ^2^Orthopaedic Surgery Department, Biel, Switzerland; ^3^Orthopaedic Surgery Department, Privatklinik Linde, Biel, Switzerland; ^4^Infectious Diseases Department, La Tronche, France; ^5^Orthopaedic Surgery Department, Lyon, France

## Abstract

We hereby report the case of a primary acromioclavicular septic arthritis caused by *Veillonella parvula*. This bacteria is rare as a pathogen but is well known as a commensal of the lungs, vagina, mouth, and gastrointestinal tract of humans. However, it may turn as an opportunistic pathogen. It was isolated in blood culture and confirmed in biopsy specimen. The patient had complete recovery after surgical washout with second look at postoperative day two and targeted antibiotic treatment.

## 1. Introduction

Native joint septic monoarthritis incidence accounts for 4 to 10 per 100 000 patients per year in Western Europe [1]. They are mostly caused by *Staphylococcus aureus*, then *Staphylococcus epidermidis*, streptococci, and Gram-negative rods [2]. Acromioclavicular joint (ACJ) septic arthritis is rare: 32 cases were reported in the literature so far [3–5].

*Veillonella parvula* is an anaerobic biofilm-forming commensal bacteria in the lungs, vagina, mouth, and gastrointestinal tract of humans. It can be an opportunistic pathogen [6].

We report the case of a patient who had a primary septic acromioclavicular arthritis caused by *Veillonella parvula*.

## 2. Case Presentation

A 43-year-old male patient with diabetes mellitus and psychic disorders, institutionalized in a home for chronic alcohol abuse, presented himself in the emergency department because of a painful right shoulder with redness for two days. He reported no fever nor shivering. He denied previous trauma or pain over his right ACJ.

The physical assessment of the right shoulder pointed out redness associated with tenderness and local warming over the acromioclavicular region. The passive and active rotations were painless, but the abduction and elevation over 70 degrees and the crossover elicited pain in the acromioclavicular region. There was no vascular nor neurological deficit. The rest of the body examination was normal except a poor dental hygiene.

**Figure 1:**
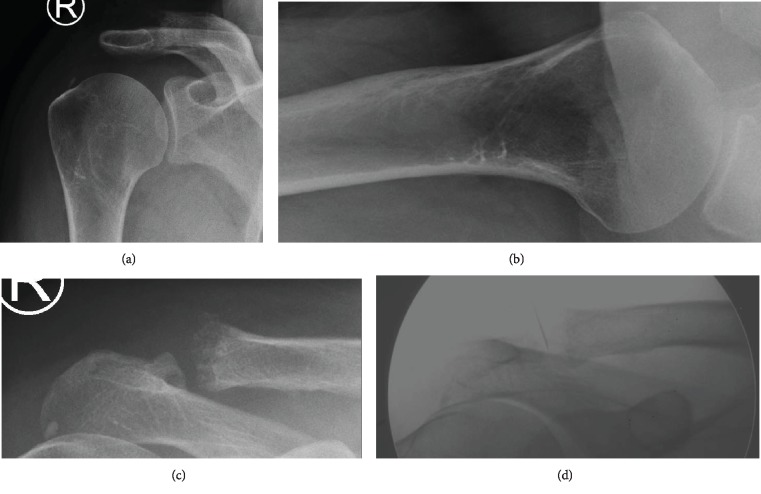
X-ray and fluoroscopic views of the right shoulder. (a) Anterior-posterior view. (b) Axial view. (c) Zanca view. (d) Acromioclavicular small-needle aspiration under fluoroscopic guidance.

Biological findings showed an inflammatory syndrome, with a white blood cell count (WBCC) of 10.6 G/l and a C-reactive protein (CRP) level of 136 mg/l. Anterior-posterior, axial, and Zanca view x-rays showed an osteolysis of the distal clavicle (Figure 1).

We performed blood cultures and a small-needle aspiration of the ACJ, using fluoroscopic guidance (Figure 1). The finding was a purulent material. Gram stain was negative finding only leukocytes because of little amount of material for the analysis.

**Figure 2:**
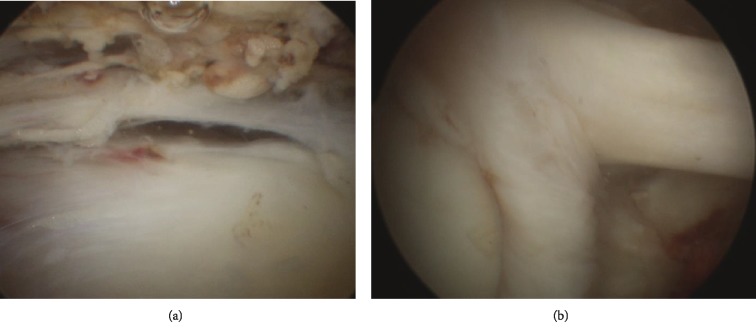
Arthroscopic views of the right shoulder. We can notice the absence of inflammatory aspect of the tissues. (a) Subacromial space. (b) Glenohumeral space with the long head of the biceps brachii.

Then, the patient was informed for consent of a shoulder arthroscopy with biopsies of the glenohumeral joint and subacromial space (Figure 2), followed by an open surgical washout with capsule and clavicle biopsies and partial distal clavicular resection. Intraoperative arthroscopic findings showed no inflammation of the glenohumeral joint and subacromial space (Figure 2). The open approach on the ACJ showed pus and local inflammation.

After sampling, the patient received an empiric intravenous antibiotic treatment with amoxicillin-clavulanic acid 2.2 g 3 times per day. An open second look of the ACJ was performed after 48 hours and showed no sign of local inflammation.

**Figure 3:**
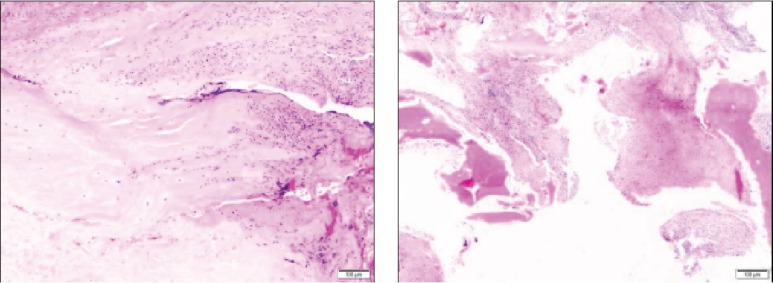
Histologic findings. (a) A capsule biopsy, with massive acute inflammation compatible with an infection. (b) A distal clavicle bone sample, with signs of chronic inflammation associated with mild acute inflammation. There was no malignancy.

No bacterial growth was observed in the glenohumeral and subacromial biopsies (0/4). After enrichment, *Veillonella parvula* was identified in the acromioclavicular and distal clavicle biopsies (6/6), as well as in the needle aspiration (1/1). Additionally, the biopsy confirmed the presence of an acute inflammation compatible to a local infection (Figure 3). However the blood culture remained negative (0/2). The resistance chart showed sensitivity of *Veillonella parvula* to amoxicillin-clavulanic acid, imipenem, clindamycin, and metronidazole.

The patient was hospitalized and treated with intravenous amoxicillin-clavulanic acid 2.2 g 3 times daily for 10 days. The laboratory controls showed progressive decrease of the inflammatory syndrome. He was discharged from the hospital after 20 days. Then, he received a 5-week additional oral treatment with clindamycin 600 mg 3 times daily.

He reported no fever during the therapy time and having no pain. He could freely move his right shoulder and presented no limitation in his own work. Then, he was discharged, and no further investigation was needed. At the 2-year follow-up consultation, the patient was asymptomatic, without any limitation nor pain in shoulder motion, and the scars were closed and without inflammation.

## 3. Discussion

The knee, hip, and shoulder are the most common joints suffering from septic arthritis, because they have the richest blood supply and are therefore more at risk [7]. All synovial joints might be infected. Hematologic dissemination is the cause in most of the cases. The ACJ is less vascularised and is rarely infected. The most frequent microorganisms are the same for ACJ: *Staphylococcus aureus* and streptococci [8–10]. A failure to treat could be complicated with osteoarthritis or sepsis and might even be lethal [7]. Usually, the small-joint septic arthritis occurs because of a deficient immune system [5].

To our knowledge, primary septic ACJ infection has been reported 32 times. In 2014, Hashemi et al. described a case from bilateral acromioclavicular septic arthritis with a literature review [3]. They showed a total number of 30 cases. After 2014 and until today, two additional cases were reported in the literature [4, 5]. In most of the cases, the patient presented comorbidities such as diabetes mellitus, immune deficiencies, and intravenous drug abuse. Most of the patients were successfully treated with intravenous antibiotics in each case and additional surgical procedure for most of them (joint aspiration, washout, and surgical debridement). The pathogens were *Staphylococcus aureus* for half of them (16/32), then group B streptococcus (3/32), *Streptococcus viridans* (2/32), *Streptococcus pneumoniae* (2/32), Salmonella, *Haemophilus parainfluenzae*, *Mycobacterium avium* and *tuberculosis*, and finally *Ochrobactrum anthropi* for only one case each. Three cases did not find any causal bacteria.

Hirai et al. made a review of the English literature on PubMed from 1976 to October 2015 [11] about infections caused by *Veillonella* spp. in humans; 31 cases were found. Five of them were with a *Veillonella parvula* infection of the musculoskeletal system (2 osteomyelitis [12, 13], 1 discitis [14], and 1 sacroiliitis [15]). The current case is the first of ACJ septic arthritis caused by *Veillonella parvula*.

All the findings in this case concluded to the involvement of the ACJ to be isolated. An osteomyelitis of the lateral clavicle might have contaminated the ACJ by contiguity. The other hypothesis is that the septic arthritis occurred first and produced inflammatory changes in the lateral clavicle. The pathogen was likely blood driven, from the mouth, because of the poor dental hygiene of the patient. The ACJ and distal clavicle had already degenerative changes supported by X-ray and histology, but the patient suffered no complain until the infection occurred. The chronic changes may have favoured a slow progressive infection, so without fever, unlike many other clinical cases. A standard treatment of septic arthritis with two surgical washouts and antibiotics was effective to cure the patient. A 2-week intravenous antibiotic therapy followed by 4 weeks of oral antibiotics seemed to be a valuable option to treat *Veillonella parvula* arthritis.
